# Complete Genome Sequence of *Serratia* sp. Strain CC119, Associated with Inner Cotton Boll Rot via Insect Vector Transmission

**DOI:** 10.1128/MRA.01077-20

**Published:** 2020-12-10

**Authors:** Enrique G. Medrano, Timothy P. L. Smith, James P. Glover, Alois A. Bell, Michael J. Brewer

**Affiliations:** a Insect Control and Cotton Disease Research Unit, U.S. Department of Agriculture, Agricultural Research Service, College Station, Texas, USA; b U.S. Meat Animal Research Center, U.S. Department of Agriculture, Agricultural Research Service, Clay Center, Nebraska, USA; c Department of Entomology, Texas A&M AgriLife Research and Extension Center in Corpus Christi, Corpus Christi, Texas, USA; University of Maryland School of Medicine

## Abstract

*Serratia* species are Gram-negative bacteria that can infect both animals and plants. The annotated genome presented is the first for a *Serratia* sp. strain (called CC119) that is a cotton boll pathogen. The opportunistic strain is associated with the boll-piercing-sucking insect *Creontiades signatus*.

## ANNOUNCEMENT

The *Enterobacteriaceae* genus *Serratia* occurs in various habitats (1). Certain species, including Serratia marcescens, can attack human or crop hosts. Phytopathogenic *Serratia* sp. strains infect cultivated crops such as alfalfa, watermelon, and squash (2, 3). Cotton is a globally grown commodity that is plagued by infestations by several pests, including cotton seed feeders. For example, the southern green stink bug (*Nezara viridula*) is a vector of boll rot pathogens (4). The complete genome of one of the pathogens associated with the stink bug and boll rot was reported (5). Recently, another significant cotton pest (the verde plant bug [*Creontiades signatus*]) was shown to transmit *Serratia* sp. strain CC119 into cotton bolls, resulting in disease (6). In this study, the annotated whole genome was generated in an effort to identify predicted products potentially involved in cotton boll infections.

*Serratia* sp. strain CC119 was isolated from a diseased cotton boll that had been infested with the verde plant bug. The cotton boll was collected from a field-grown plant at a Texas A&M AgriLife Research and Extension Center plot in Corpus Christi, TX (GPS coordinates: 27.7765, −97.5621). Bolls were surface sterilized using 5% sodium hypochlorite, rinsed in sterile distilled water (three times), and then plated onto Luria-Bertani agar (LBA) (Difco Laboratories, Detroit, MI). Following a 2-week incubation at 27°C under aerobic conditions, plates were observed for growth. Numerically predominant colonies were purified and analyzed for infectivity based on Koch’s postulates, using greenhouse-grown bolls. Disease-causing representatives were Gram stained and putatively identified to the genus level using standard API 20E test strips (bioMérieux, Inc., Hazelwood, MO). For sequencing, the strain was cultured for 16 to 18 h on LBA using the conditions described above. A DNeasy kit (Qiagen, Hilden, Germany) was used to extract genomic DNA, and sequencing was performed on a Pacific Biosciences Sequel instrument as suggested by the manufacturer, using the SMRTBell Express template preparation kit v2.0 without size selection. The library was sequenced using a 10-h movie collection time with a single-molecule real-time (SMRT) Cell 1M v3, producing 674,449 reads and a unique molecular yield of 7.6 Gb, with a subread mean length of 10.8 kb (*N*_50_, 18.2 kb). The read quality control, error correction, and adapter trimming functions were based on the Microbial Assembly application of SMRT Link v9.0.0.92188. The genome was assembled in SMRT Link v9.0.0.92188 using the Microbial Assembly protocol and default settings, with the expected genome size set at 5 Mb. The genome was determined to be complete by mapping the reads back to the circularized genome using minimap2 and verifying reads that spanned the junction. The Microbial Assembly application of SMRT Link performs circularization and trimming and rotates the assembly to place the origin of replication at the beginning of the final linearized assembly. The output assembly consisted of two contigs, i.e., the chromosome of 5.1 Mb and an extrachromosomal plasmid of 123 kb; the two contigs had approximately 1,320× and 1,198× coverages, respectively, as reflected by a mean quality value of 92 for both. A computational annotation using the Prokaryotic Genome Annotation Pipeline (PGAP) at the NCBI was conducted and curated.

A total of 4,914 genes, including 4,784 coding DNA sequences with 22 rRNA operons, 89 tRNAs, and 18 noncoding RNAs, were predicted. The GC contents of the circular chromosome and the plasmid were 59% and 53%, respectively. Putative genes associated with host infection included a type IV secretion system, TonB-dependent receptor, porin, and invasin. These identified pathogenicity and virulence factors may be involved in boll disease.

### Data availability.

Raw sequencing data were deposited in the NCBI SRA database (accession number SRR12563834), with broader information available under BioProject accession number PRJNA487218. This whole-genome shotgun project was deposited in DDBJ/EMBL/GenBank with the accession numbers CP060276.1 and CP060277.1.
